# New possibilities: the LockDown device for distal clavicle fractures

**DOI:** 10.1016/j.jseint.2020.08.021

**Published:** 2020-10-15

**Authors:** Leanne S. Blaas, Maayke N. van Sterkenburg, Annick M. de Planque, Robert J. Derksen

**Affiliations:** a Department of Trauma Surgery, Zaandam Medical Center, Zaandam, The Netherlands; b Department of Trauma Surgery, Noordwest Ziekenhuisgroep, The Netherlands

**Keywords:** Distal clavicle fracture, LockDown device, distal clavicle resection, AC joint, AC dislocation, CC ligament

## Abstract

**Background and hypothesis:**

The majority of distal clavicle fractures are displaced fractures and constitute a treatment challenge because they have a 30% chance of delayed union or nonunion. Although several options for surgical reconstruction have been described, in patients with a comminuted and/or small distal fragment, these reconstructive options have proved to be prone to failure. Moreover, secondary surgery for removal is necessary in most cases. We hypothesized that the LockDown device, a braided synthetic ligament device, combined with resection of the distal fracture fragment is a suitable alternative in specified patients with distal clavicle fractures.

**Methods:**

Eleven patients with distal clavicle fractures were treated with distal fracture resection and the LockDown procedure. All patients underwent regular follow-up with data collection; additionally, 7 were assessed at 1-year follow-up according to the study protocol. On the basis of radiography, these patients had a clear coracoclavicular ligament disruption and subsequent cranial dislocation of the medial fragment. Regular follow-up was performed at 6 weeks, 3 months, and 6 months. Control radiographs were taken at 3 and 6 months. Furthermore, the 7 enrolled patients were assessed at 1 year, when the Disabilities of the Arm, Shoulder and Hand score, Constant shoulder score, Nottingham Clavicle Score, and range of motion were recorded. Residual pain was ascertained by a visual analog scale score.

**Results:**

In total, 11 patients were treated with distal clavicle resection and the LockDown procedure. Eight patients underwent surgery within 3 weeks after presentation at the emergency department. The other 3 patients were operated on after a trial of conservative treatment (due to persisting pain and delayed union). None of the patients had postoperative complications. At 3 months, 9 of the 11 patients had made a full recovery.

**Discussion:**

All 11 patients had good short-term clinical outcomes. None showed acromioclavicular instability. Furthermore, secondary surgery was avoided, and hardware complications did not occur. In low-demand patients or patients with a high risk of nonunion, this technique may be a favorable alternative to other known techniques.

Distal clavicle fractures account for 17%-30% of all clavicle fractures.^7^^,^^9^^,^^14^ Of these, 51%-55% are significantly displaced fractures indicative of coracoclavicular (CC) ligament rupture.^7^^,^^9^^,^^14^ Furthermore, there is a 30% chance of delayed union or nonunion.^14^^,^^15^^,^^18^ Clavicular fractures have a bimodal age distribution. The first peak occurs in young active adult men, and the second peak occurs in elderly women with osteoporosis. Distal-end fractures occur more commonly in the latter age group.^17^ The acromioclavicular (AC) joint articulation anchors the clavicle to the scapula. Horizontal and vertical stability of the AC joint is required. Static restraints include the AC, CC, and coracoacromial ligaments (Fig. 1). The AC ligaments and joint capsule provide horizontal translation. The CC ligament is divided into 2 portions: the posteromedial conoid and the anterolateral trapezoid. The conoid prevents vertical translation of the distal clavicle, and the trapezoid confers axial stability. More dynamic restraints of the AC joint include the deltoid, trapezius, and serratus anterior musculature. Movement in the AC joint includes rotation (5°-8° with forward elevation and abduction of the arm) and translation in the anteroposterior and superoinferior directions. Additionally, the AC joint serves as the pivot point for scapular (acromial) protraction and retraction.^20^

**Figure 1:**
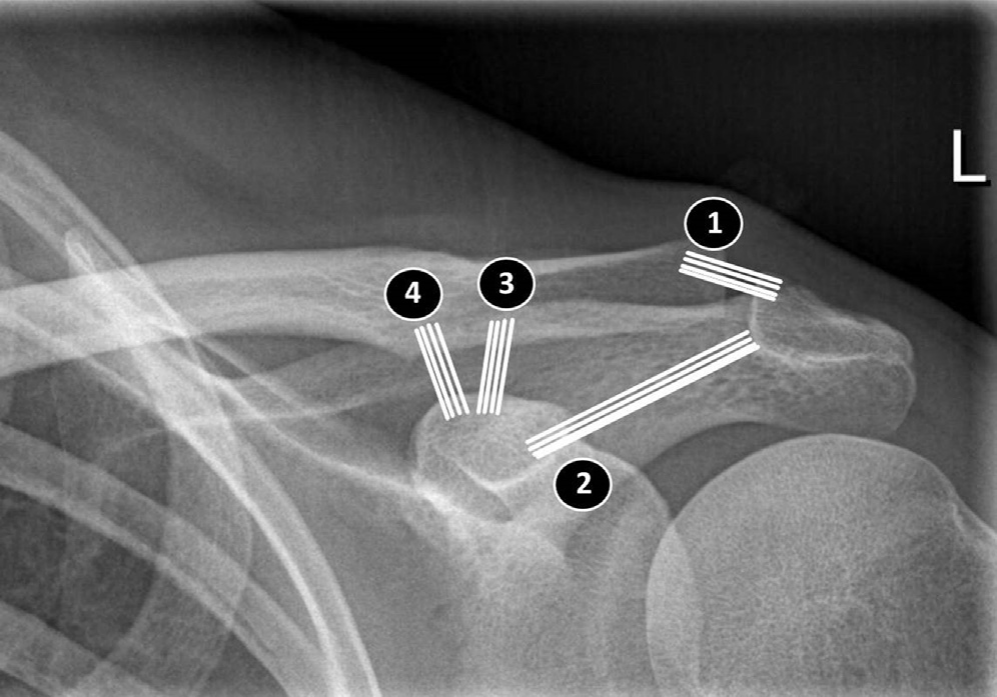
Schema of ligaments: acromioclavicular ligament *(1)*, coracoacromial ligament *(2)*, and coracoclavicular ligament with trapezoid *(3)* and conoid *(4)* parts. *L*, left.

Distal clavicular fractures are classified according to Neer^12^ (Fig. 2). In elderly patients, as well as smokers and patients with comorbidities such as diabetes, the likelihood of nonunion and consequent chronic pain and disability is more pronounced in unstable distal clavicle fractures (eg, fractures in which the medial fragment is not stabilized by the CC ligament).^6^^,^^10^ When conservative treatment fails and a painful nonunion remains or if surgery is indicated because of severe dislocation of fragments caused by disruption of the CC ligament, there are several options for reconstruction. The most common procedure is open reduction and internal fixation of the fracture with a combination small- and mini-fragment distal clavicle plate containing multiple locking mini-fragment options on the lateral aspect of the implant. Nevertheless, in patients with a comminuted distal fragment or with a fragment < 2 cm, especially when bone stock is poor, fixation might not be stable enough. Implant failure or nonunion may occur. Furthermore, in a biomechanical study, the distal clavicle plate showed less construct strength compared with cortical button fixation.^23^ The hook plate is a well-known option for comminuted or small distal clavicle fragments; however, it has been reported to be painful until the mandatory removal of hardware.^10^ In addition, the hook passes through the AC joint, making it prone to cause damage to the cartilage with a subsequent risk of symptomatic arthritis. Moreover, abduction is allowed to only 90°, owing to the possibility of cuff injury or wear of the acromion due to friction of the subacromial hook. Secondary surgery for removal is necessary in most cases because of hook migration into the acromion and pain.^11^ The hook plate has been associated with high failure rates such as implant failure, reoperation, and redislocation after removal.^19^

**Figure 2:**
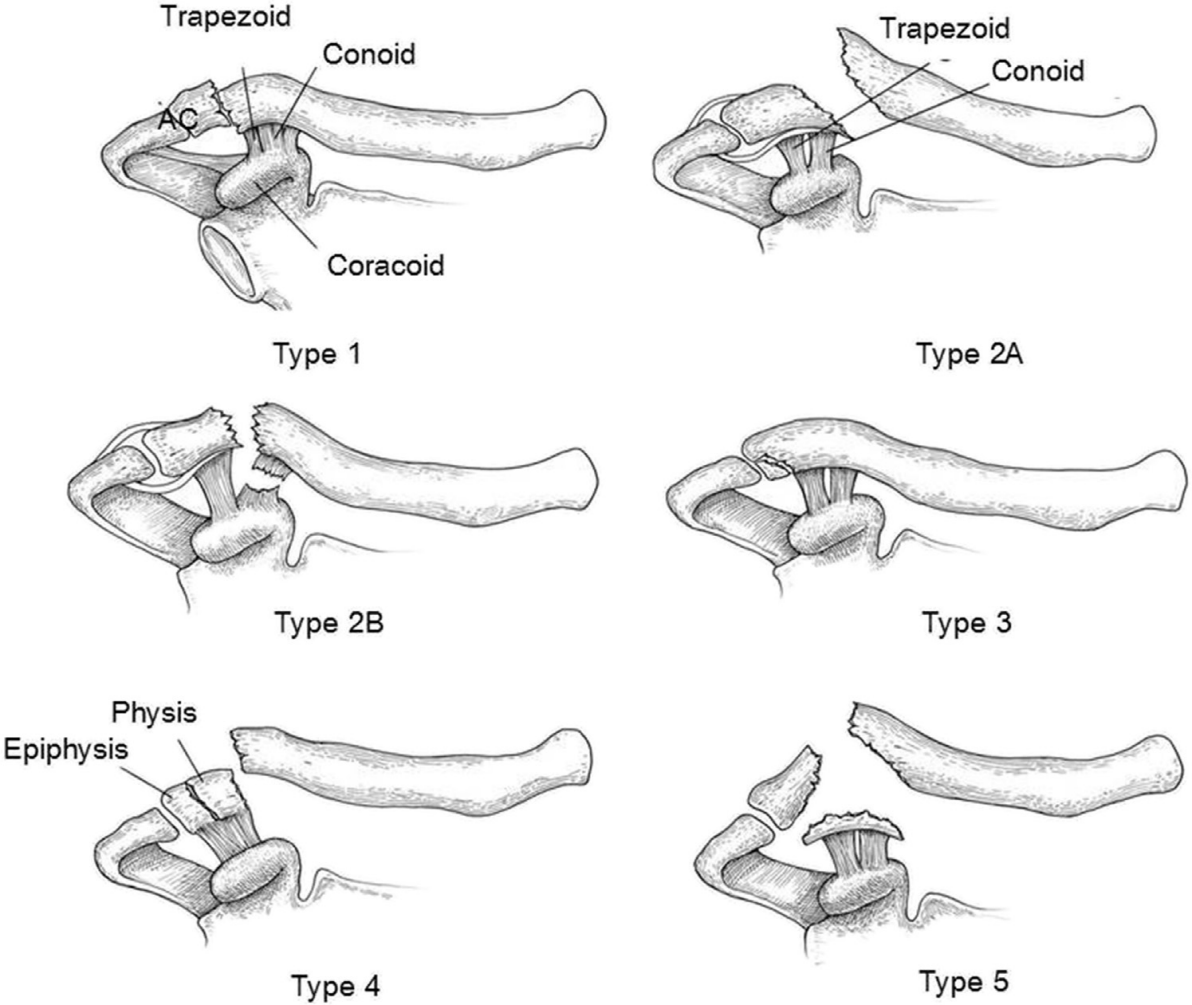
Neer classification. Type 1 is a fracture lateral to the coracoclavicular ligament, in which the conoid and trapezoid remain intact, with minimal displacement. Type 2A is a fracture medial to the coracoclavicular ligament, in which the conoid and trapezoid remain intact, with medial clavicle displacement. Type 2B is a fracture that occurs between or lateral to the coracoclavicular ligaments, in which the conoid is torn and the trapezoid may be intact, with medial clavicle displacement. Type 3 is an intra-articular fracture, in which the conoid and trapezoid remain intact, with minimal displacement. Type 4 is a physeal fracture in an immature skeleton, in which the conoid and trapezoid remain intact, with lateral clavicle displacement. Type 5 is a comminuted fracture, in which the conoid and trapezoid remain intact, with medial clavicle displacement.^1^*AC*, acromioclavicular.

We hypothesized that the LockDown device (LockDown Surgical, Chanhassen, MN, USA), a braided synthetic ligament device, combined with resection of the distal fracture fragment would be a suitable alternative in older patients with distal clavicle fractures with CC ligament disruption, Neer type 2 (Fig. 3), and in patients with a painful nonunion of fractures of all Neer types (Fig. 4). We report on 11 cases in which this procedure was performed.

**Figure 3:**
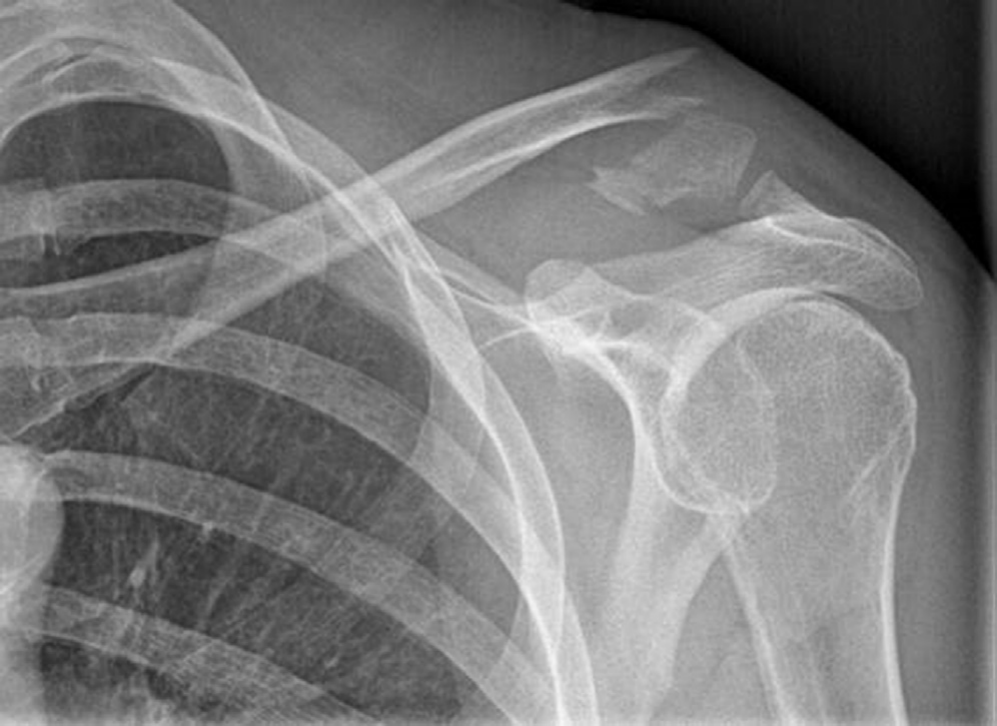
Radiograph of patient with Neer type 2 fracture.

**Figure 4:**
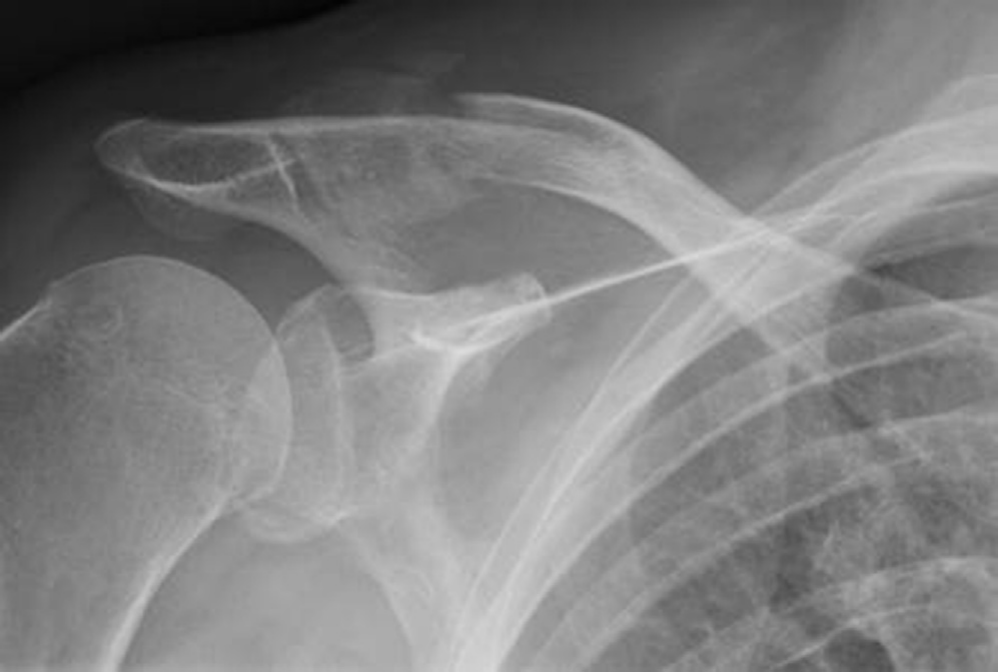
Radiograph of patient with painful nonunion.

## Materials and methods

**Figure 5:**
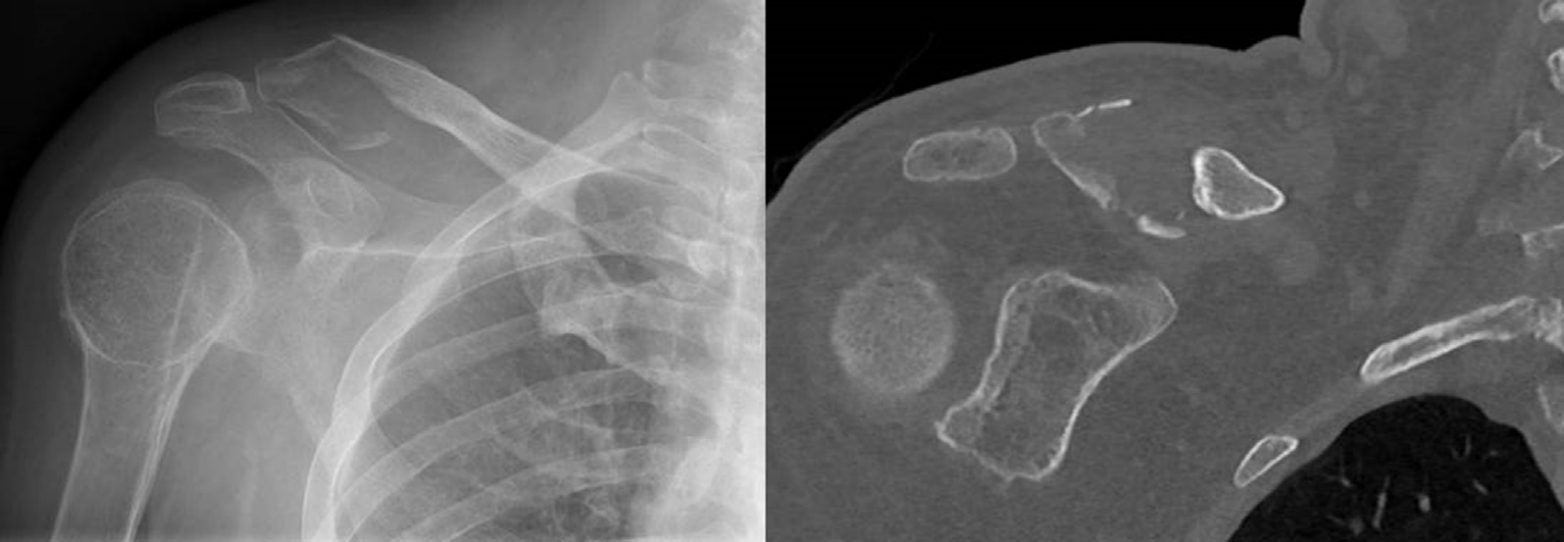
Imaging of comminuted distal clavicle fragment in case 1.

Since 2016, 11 patients have been treated with distal fracture fragment resection and the LockDown procedure. The indication for this treatment was based on the fracture configuration on radiographs (Neer classification). Patients with CC ligament disruption and considerable cranial dislocation of the distal fragment were selected. When the size or amount of comminution of the distal fragment was unclear, a computed tomography scan was performed. In cases deemed unstable (Neer types 2, 4, and 5), when the distal fragment was <3 cm in size and osteoporotic, or when the fragment was severely comminuted (Fig. 5), this technique was considered suitable. Furthermore, delayed union or persistent pain after conservative treatment was also considered an indication for resection and LockDown fixation. In 8 patients, semi-elective surgery (within 3 weeks of injury) was planned, whereas 3 patients were treated after failed conservative treatment.

**Figure 6:**
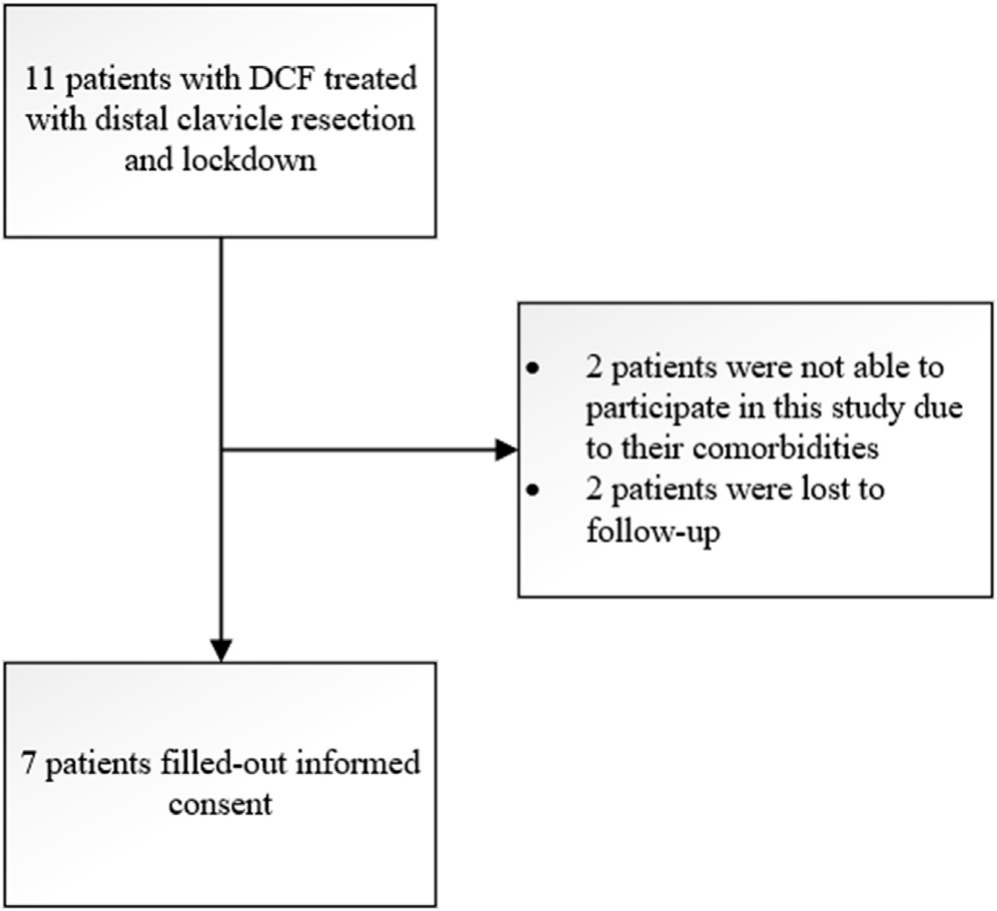
Inclusion of patients. At the time of inclusion, 2 patients were not able to participate in the study because they were undergoing treatment for malignancies. *DCF*, distal clavicle fracture.

In 2019, we approached all 11 patients with distal clavicle fractures treated with the LockDown device to obtain final measurements. Of these, 7 patients agreed to participate and signed the informed consent form (Fig. 6). Of the other 4 patients, 2 were lost to follow-up and 2 were undergoing treatment for newly diagnosed malignancies and were not able to participate because of their treatment schedule. The 7 aforementioned patients answered 3 questionnaires in an interview style: Disabilities of the Arm, Shoulder and Hand score; Constant shoulder score; and Nottingham Clavicle Score. Furthermore, the visual analog scale (VAS) score was assessed, and range of motion was measured with a protractor. Other patient characteristics recorded were age, smoking status and/or physical performance level, and comorbidity.

**Figure 7:**
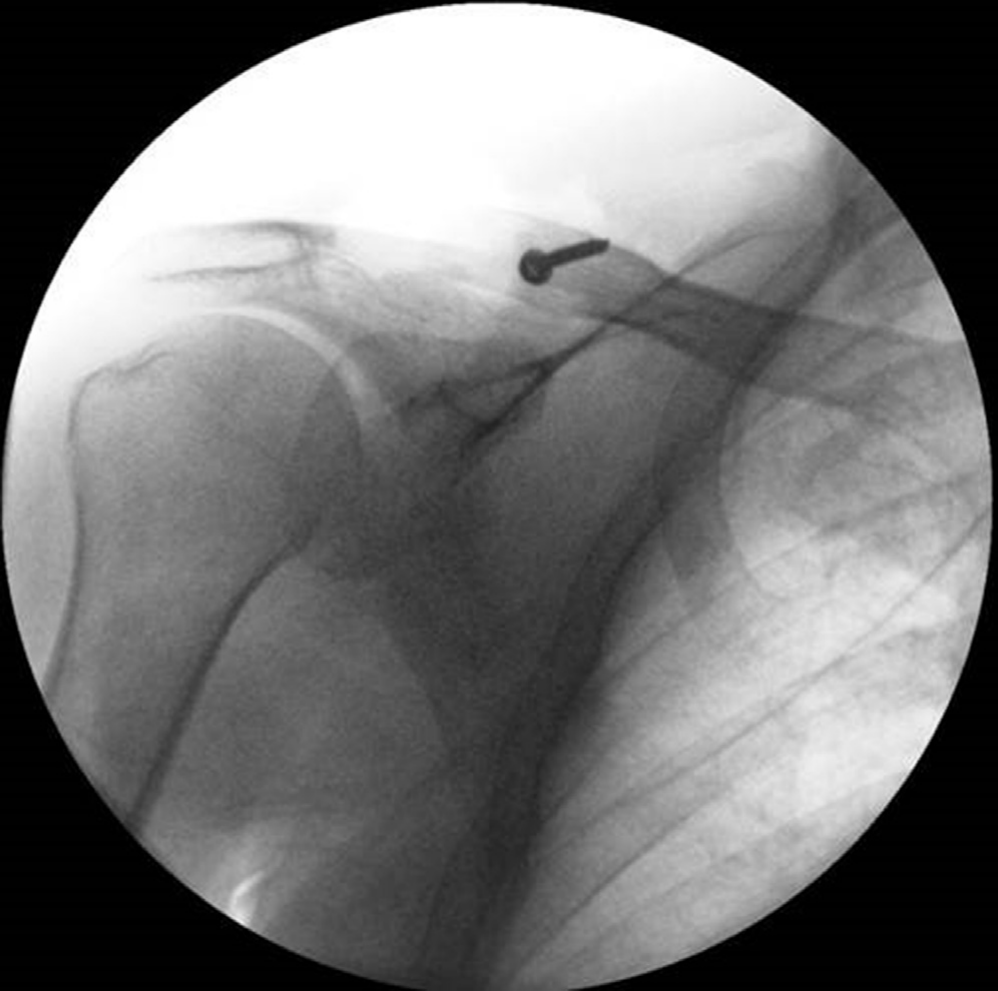
Fluoroscopic image after placement of LockDown device.

**Figure 8:**
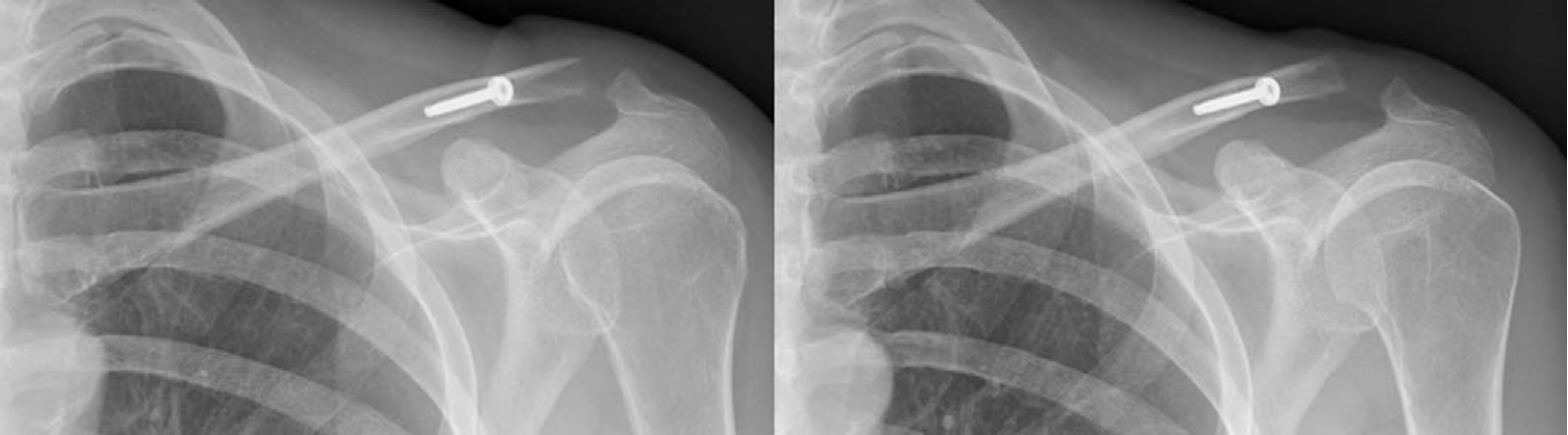
Radiographs at 6 weeks (left) and 3 months (right).

Statistical analyses were executed by descriptive statistics. IBM SPSS software (version 26; IBM, Armonk, NY, USA) was used.

### Surgical procedure

The LockDown device is a braided polyester augmentation device originally used to treat AC joint dislocations.^8^^,^^21^ All patients received general anesthesia; 6 of the 11 patients received a complementary plexus block. All patients were placed in the beach-chair position. After disinfection and sterile draping, a longitudinal incision was made from the distal clavicle to the coracoid process. The anterior portion of the deltoid muscle was carefully dissected off the distal clavicle and fringed for later reattachment. The distal fracture part was removed and the coracoid base identified. The measurement device was used in the typical manner, after which the appropriate-sized polyester ligament was passed through. A 2.5-mm hole was drilled in the clavicle from anterolateral to posteromedial, after which the ligament was attached with a 3.5-mm non–self-tapping screw of measured length (+4 mm considering the caliber of the ligament and washer). Reduction of the clavicle in relation to the acromion was checked using fluoroscopy (Fig. 7). The anterior segment of the deltoid muscle was reattached, covering the screw head, to diminish postoperative pain from the implant and screw. Both the subcuticular tissue and the skin were closed with absorbable sutures. A compressive dressing was applied for 2 days.

### Postoperative management

The arm was rested in a sling for 1-2 weeks for wound healing, allowing rotational shoulder exercises. Subsequently, a 4-week period of passive and active non–weight-bearing motion in the horizontal plane was allowed, preferably guided by a shoulder physical therapist. At 6 weeks, patients returned to the outpatient clinic. Routine radiography was performed to evaluate congruency of the AC joint and to ensure there was no implant failure (Fig. 8). At 6 weeks, full range-of-motion exercises were allowed. At 3 months, return to normal activities was permitted. At 6 months, final follow-up was performed.

## Results

The patient characteristics of all patients treated with distal clavicle resection and the LockDown procedure are shown in Table I. The age of the patients ranged between 24 and 76 years, with a median age of 62 years.

**Table I:** Patient characteristics

	Sex	Age, yr	Comorbidity	ASA class	Acute (≤3 weeks) or delayed (>3 weeks)	Fragment size, mm	Neer classification
Case 1∗	M	62	Paresis ipsilateral	2	Acute (1 week)	28	2b
Case 2∗	F	61	—	1	Acute (1 week)	16	2b
Case 3∗	F	76	Hypertension, angina pectoris	2	Delayed (6 weeks)	13	2b
Case 4∗	M	60	Heavy smoker	2	Delayed (13 weeks)	30	1
Case 5	F	67	Hypothyroidism	2	Acute (2 weeks)	22	2b
Case 6	F	65	Heavy smoker, COPD	2	Delayed (13 weeks)	27	1
Case 7∗	M	32	—	1	Acute (2 weeks)	17	2b
Case 8∗	M	74	—	1	Acute (2 weeks)	18	2b
Case 9∗	V	75	Hypertension, angina pectoris, hypothyroidism, DVT	3	Acute (1 week)	19	2a
Case 10	M	58	Hemophilia A, type 2 diabetes mellitus, liver transplantation	3	Acute (3 weeks)	13	2b
Case 11	M	24	—	1	Acute (3 weeks)	22	2b

*ASA*, American Society of Anesthesiologists; *M*, male; *F*, female; *COPD*, chronic obstructive pulmonary disease; *DVT*, deep venous thrombosis.

∗ Included in case series.

One patient with diabetes was included in our population. Three patients used anticoagulants, and 2 were heavy smokers.

Eight patients were scheduled for surgery at presentation in the emergency department. They had clear disruption of the AC capsule and CC ligament, with superior displacement of the medial clavicle, and therefore a high likelihood of nonunion if left unstabilized. In 3 patients, surgery was performed after failed conservative treatment; due to persisting pain and delayed union, resection of the distal clavicle fragment was planned. The fragment size ranged from 13 to 30 mm. As the size of the fragment in all cases exceeded 10 mm, the clavicle was stabilized with the LockDown implant. No postoperative complications occurred. At 6 weeks, all patients complained of slight discomfort and limitation in active abduction and anteflexion. At 3 months, 9 of the 11 patients were complaint free. Two reported slight anterior discomfort at the level of the screw, and 1 patient still complained of pain. We could not relate this to the procedure. This patient had a good postoperative outcome, but after a second fall on the same shoulder, brachial plexopathy was diagnosed after consultation with a neurologist. At 6 months' follow-up, there was no change or increase in complaints in all 11 patients. The 7 patients included in the case series were assessed after 1-year follow-up (Table II). These patients had excellent Constant shoulder scores; Disabilities of the Arm, Shoulder and Hand scores; and Nottingham Clavicle Scores. The range-of-motion assessment showed very small to no differences compared with the uninjured arm.

**Table II:** Results after 1 year of follow-up

	No. of patients or median (interquartile range)
Patients	7
Sex: female	3
Fracture side: right	4
Age, yr	62 (61-75)
No. of planning procedures	2 (1-6)
Fragment size, mm	18.6 (16-28)
VAS score	1 (0-4)
CSS	9.5 (1.5-14.5)
DASH score	3.40 (1.7-22.4)
NCS	92 (76.0-100)
Anteflexion, °	
Fractured side	156.5 (139-180)
Non-fractured side	156.5 (151.8-180)
Abduction, °	
Fractured side	160 (139-177)
Non-fractured side	171 (146.5-178.5)
External rotation, °	
Fractured side	48.5 (33-56.5)
Non-fractured side	50 (45.25-70.5)

*VAS*, visual analog scale; *CSS*, Constant shoulder score; *DASH*, Disabilities of the Arm, Shoulder and Hand; *NCS*, Nottingham Clavicle Score.

## Discussion

Eleven patients with Neer type 2 distal clavicle fractures or painful delayed union of distal clavicle fractures (2 Neer type 1 and 1 Neer type 2) were treated with distal fragment resection and LockDown stabilization. They have shown good short-term clinical outcomes. Secondary surgery following either discomfort due to the device or hardware complications has not been necessary thus far (median follow-up period, 27.3 months), in contrast to the frequent necessity for secondary surgery after distal plating and hook plate procedures.^11^^,^^19^ Furthermore, because of the distal clavicle resection, necrosis of the cartilage and an incongruent articulation between the acromion and clavicle are averted; thereby, osteoarthritis is prevented. Intra-articular (especially incongruent) distal clavicle fractures and/or distal clavicle fractures treated with a hook plate are prone to osteoarthritis. Nonunion, owing to, for example, smoking and diabetes, is prevented. Although the largest fragment excised in our study was 28 mm, none of the patients had signs of postoperative AC instability, whereas over-resection has been described in distal clavicle resection for AC osteoarthritis.

Although the AC capsule provides horizontal stability, Mazzocca et al^10^ stated that anatomic reconstruction procedures involving both the conoid and trapezoid ligaments appear to have the ability to control anteroposterior translation without the need to reconstruct the AC capsular ligaments. This gave us reason to believe that resection of the distal clavicle, even with segments slightly larger than 8 mm, would be permitted because trapezoid and conoid function would be taken over by the synthetic ligament.

However, Gokkus et al^6^ and Boehm et al^4^ stated that in cases of a resection of >5-10 mm, AC joint instability can occur. This assertion was supported by Pandhi et al,^13^ who found that the anteroposterior load to clinical failure of the AC joint after 5 mm of resection from the distal clavicle (and medial acromion) is significantly greater than that with 10 mm of resection of the distal clavicle alone. Moreover, Eskola et al^5^ found that patients with resection of >10 mm, with osteoarthritis or traumatic separation of the AC joint, experienced more pain. When a more limited resection of 5 mm is performed and the inferior capsule is preserved, Gokkus et al found that cutting the AC ligament did not cause symptomatic instability. In their anatomic study of 36 shoulders, Boehm et al found that resection of 10 mm of the distal clavicle detaches an average of 8% of the trapezoid ligament; moreover, with 20 mm, this increased to 60%. Therefore, they hypothesized that resection of >10 mm may lead to AC joint instability. According to Blazar et al,^3^ the amount of AC instability was directly correlated to the VAS pain score but did not correlate to the apparent joint space seen on radiographs after surgery.

When instability occurs after over-resection, there are a variety of surgical options with modifications to Weaver-Dunn reconstruction, including the addition of CC stabilization with a screw, suture, or graft.^20^ However, in our procedure, possible over-resection causing CC instability in grade Neer 1, 2a, or 3 nonunions is directly prevented by using the LockDown device as a stabilizing device. In type 2b fractures, the CC ligaments are already disrupted. They are surgically stabilized by the LockDown device, and the distal fragment is resected, with a good outcome and a low VAS score of 1-4.^16^

With resection of the distal clavicle and use of the LockDown device, the biomechanical function of the AC joint is not restored. This may hypothetically cause a 5°-8° reduction of forward elevation and abduction of the arm as compared with the other side. This is supported by the results of our case study. The minimal functional loss is, in our opinion, acceptable in a lower-demand patient group, but it should be taken into consideration in younger patients and athletes. If dyskinesis of the scapula was at all present, it was not evident during the regular follow-up of the outlined patients. However, we did not specifically test scapular function, and it is possible that subtle dyskinesis was missed. As suggested in the literature addressing this issue, physiotherapy is usually sufficient in compensating for subtle scapular dyskinesis. Because all patients received physiotherapy after surgical treatment, patients learned how to use and train the slightly altered mobility in case of subtle dyskinesis to obtain a normal functional outcome.^16^

In an earlier study in which the surgical procedure was similar—although performed in 2 steps and in patients with chronic instability—Baxter et al^2^ provided supporting evidence. In their case series on 13 patients with AC joint stabilization for instability following distal clavicle excision with a synthetic ligament, good results were obtained. Full resolution of symptoms was not reached, hypothetically owing to the chronicity of the patients' symptoms and multiple previous procedures.

Although our study focuses on distal clavicle fracture segment resection and stabilization by the LockDown device in patients with distal clavicle fractures, other studies have shown the effectiveness of the LockDown device in patients with AC dislocation.^22^ Wright et al^22^ reported outcomes in 21 patients undergoing AC stabilization with the braided polyester prosthetic ligament for Rockwood type 3 dislocations. The outcomes were good at a mean follow-up of 30 months, but the mean abduction power on the operated side was 82% (range, 31%-97%) of that on the normal side.

Some surgeons are reluctant to use the ligament as it does not provide exact anatomic reconstruction. Careful dissection is of major importance. The dissection and LockDown device should leave the coracoacromial ligament intact by tunneling the device posterior to this ligament. Placing the LockDown device too medially across the clavicle will leave a craniocaudal dislocation, although it will still stabilize the joint. Placing the LockDown device too distally will result in forward translation of the clavicle. Pulling the clavicle too far caudally (over-tightening) may cause screw cutout. Meticulous technique is mandatory. Furthermore, early postoperative mobilization may reduce stiffness and the chance of early adhesive capsulitis.

To our knowledge, no studies have described the use of the LockDown device for an indication other than pure AC joint disruption. In low-demand patients with a high risk of nonunion and persisting pain and in patients with comminuted or osteoporotic distal fragments, distal clavicle fragment resection with LockDown device stabilization may be a suitable alternative to osteosynthesis or hook plate fixation. Obviously, a prospective comparative study with a longer follow-up would be necessary to confirm the superiority of this treatment.

## Conclusion

In low-demand patients or patients with a high risk of nonunion, removal of the outer fracture segment in distal clavicle fractures, followed by placement of the LockDown device, appears to be a suitable treatment option for distal clavicle fractures.

## Disclaimer

The authors, their immediate families, and any research foundations with which they are affiliated have not received any financial payments or other benefits from any commercial entity related to the subject of this article.
